# Metformin Prevents Renal Fibrosis in Mice with Unilateral Ureteral Obstruction and Inhibits Ang II-Induced ECM Production in Renal Fibroblasts

**DOI:** 10.3390/ijms17020146

**Published:** 2016-01-22

**Authors:** Yang Shen, Naijun Miao, Jinlan Xu, Xinxin Gan, Dan Xu, Li Zhou, Hong Xue, Wei Zhang, Limin Lu

**Affiliations:** Department of Physiology and Pathophysiology, Shanghai Medical College, Fudan University, Shanghai 200032, China; yangshen13@fudan.edu.cn (Y.S.); 13111010002@fudan.edu.cn (N.M.); 13211010003@fudan.edu.cn (J.X.); 14211010001@fudan.edu.cn (X.G.); 14211010003@fudan.edu.cn (D.X.); lizhou@shmu.edu.cn (L.Z.); xuehong@fudan.edu.cn (H.X.); wzhang@shmu.edu.cn (W.Z.)

**Keywords:** metformin, renal fibrosis, ERK, angiotensin II, transforming growth factor-β

## Abstract

Renal fibrosis is the final common pathway of chronic kidney disease (CKD), and no effective medication is available clinically for managing its progression. Metformin was initially developed as an anti-diabetic drug and recently gained attention for its potential in the treatment of other diseases. In this study, we investigated its effects on renal fibrosis in a mouse model of unilateral ureteral obstruction (UUO) *in vivo* and in angiotensin II (Ang II)–treated renal fibroblast NRK-49F cells *in vitro*. Our data showed that UUO induced renal fibrosis and combined with the activation of ERK signaling, the upregulation of fibronectin, collagen I, and transforming growth factor-β (TGF-β). The administration of metformin inhibited the activation of ERK signaling and attenuated the production of extracellular matrix (ECM) proteins and collagen deposition in the obstructed kidneys. In cultured renal fibroblasts, Ang II increased the expression of fibronectin and collagen I and also activated ERK signaling and TGF-β in a time-dependent manner. Pretreatment of the cells with metformin blocked Ang II–induced ERK signaling activation and ECM overproduction. Our results show that metformin prevents renal fibrosis, possibly through the inhibition of ERK signaling, and may be a novel strategy for the treatment of renal fibrosis.

## 1. Introduction

Renal fibrosis is a common feature of all forms of chronic kidney disease (CKD), regardless of the initial cause of the disease [1,2,3]. It is characterized by an excessive accumulation of extracellular matrix (ECM) proteins in the kidney [4]. Accelerated ECM production alters the kidney architecture and leads to subsequent renal dysfunction, ultimately resulting in renal failure. Eventually, patients require lifelong dialysis or kidney transplantation to sustain life. There is no effective treatment for renal fibrosis, and its occurrence worldwide is gradually increasing. Thus, substantive therapeutic interventions are urgently needed to reverse renal fibrosis.

Renal fibrosis can be initiated by many pathological factors, including toxicity, ischemia, pathogenic microorganism infections, an inherited defect, or endocrine and immunological diseases [5]. When injured, interstitial fibroblasts transdifferentiate into myofibroblasts, which contribute to the accumulation of ECM proteins, such as fibronectin and collagen I [6,7]. Many studies have shown that overactivation of the intrarenal renin-angiotensin system (RAS) is involved in renal fibrosis in various CKD conditions, while transforming growth factor-β (TGF-β) is a key downstream mediator of the RAS [8,9]. Angiotensin II (Ang II) is the main effector of the RAS. Ang II stimulates the activation and transdifferentiation of fibroblasts into scar-forming myofibroblasts [10,11].

**Figure 1:**
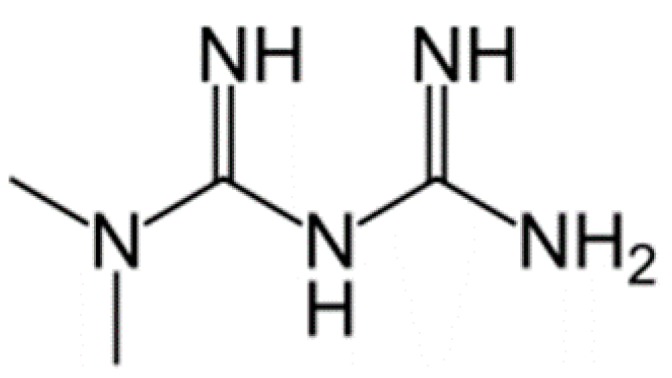
Chemical structure of metformin.

Metformin (Figure 1) is a biguanide compound used for the treatment of type 2 diabetes, and it is one of the world’s most widely prescribed drugs [12]. Due to its superior safety and relatively low risk of side effects, metformin has also been tested for its effectiveness in the treatment of other diseases. Recent reports have demonstrated its role in the treatment of cancer and cardiovascular diseases [13,14,15]. There is also evidence suggesting that metformin reduces fibrosis in nonalcoholic steatohepatitis [16,17]. Moreover, Cavaglieri *et al.* have demonstrated that the anti-fibrotic effect of metformin in renal fibrosis *in vivo* [18].

In this study, we investigated the effects of metformin on renal fibrosis both *in vivo* and *in vitro*. Unilateral ureteral obstruction (UUO) is a well-characterized animal model of renal fibrosis [19,20]. Our data showed that metformin was effective in reducing renal fibrosis in UUO mice. Moreover, metformin inhibited the ECM production induced by Ang II in renal fibroblast NRK-49F cells. Thus, our data provide a rationale for the treatment of renal fibrosis using metformin.

## 2. Results

### 2.1. Metformin Administration Attenuated Fibrosis in Obstructed Kidneys

**Figure 2:**
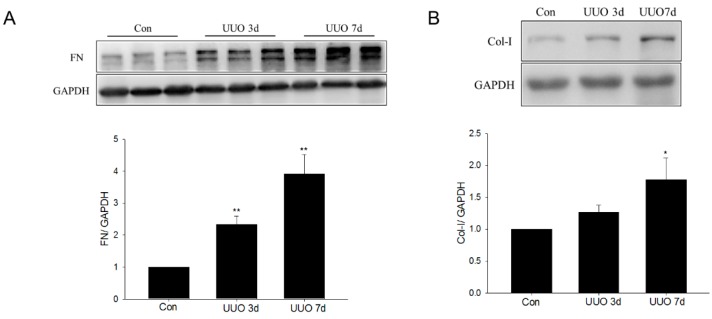

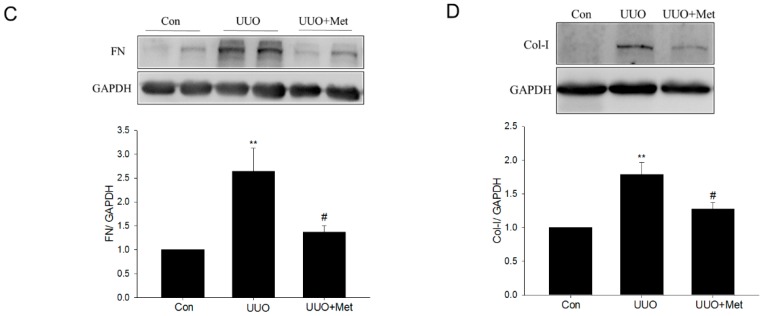
The effect of metformin on extracellular matrix (ECM) production in unilateral ureteral obstruction mice. (**A**,**B**) The time course of fibronectin and collagen I in unilateral ureteral obstruction (UUO) mice, at three and seven days, visualized by Western blotting; (**C**,**D**) The levels of fibronectin (FN) and collagen I (Col-I) were analyzed by Western blotting after metformin treatment. Data are the mean ± SEM for six animals. * *p* < 0.05, ** *p* < 0.01 compared with control (Con); ^#^ *p* < 0.01 compared with UUO with vehicle (UUO).

**Figure 3:**
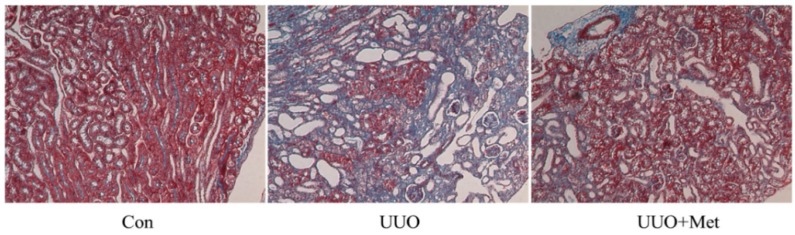
The effect of metformin on collagen production in unilateral ureteral obstruction mice. Representative sections of Masson’s trichrome-stained kidneys. Collagen was stained blue. Original magnifications, 200×.

In UUO mice, dramatic increases in the levels of ECM proteins, including fibronectin and collagen I, were observed three days after surgery and these increased further at seven days (Figure 2A,B). Metformin was administered by gavage in one group of UUO mice to determine whether it could inhibit the progression of renal fibrosis *in vivo*. The Western blot results showed that the metformin inhibited UUO-induced upregulation of fibronectin and collagen I protein expression in the obstructed kidney (Figure 2C,D). Masson’s trichrome staining showed that collagen deposition increased significantly in the interstitial area, suggesting that the UUO induced renal interstitial fibrosis (Figure 3). Metformin treatment significantly attenuated UUO-induced collagen deposition (Figure 3). Taken together, these data suggest that metformin attenuated renal fibrosis in the UUO model.

### 2.2. Metformin Inhibited UUO-Induced TGF-β Upregulation and ERK Signaling Activation

**Figure 4:**
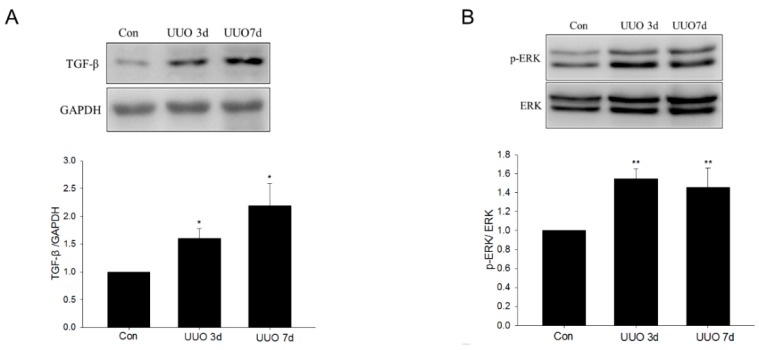

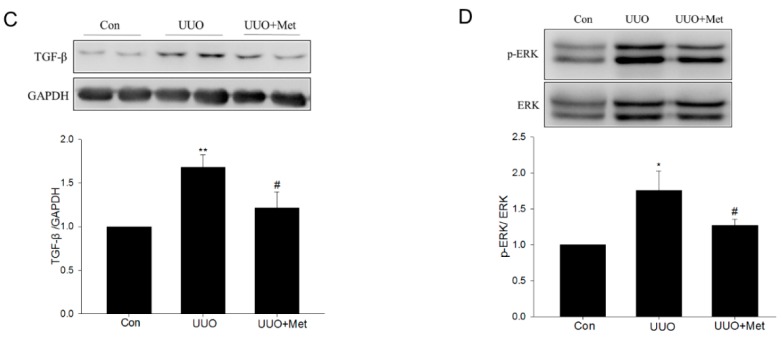
The effect of metformin on unilateral ureteral obstruction–induced transforming growth factor-β upregulation and ERK signaling activation. (**A**,**B**) The time course of transforming growth factor-β (TGF-β) and p-ERK in unilateral ureteral obstruction (UUO) mice, at three and seven days, visualized by Western blotting; (**C**,**D**) The levels of TGF-β and p-ERK were analyzed by Western blotting after metformin treatment. Data are the mean ± SEM of six animals. * *p* < 0.05, ** *p* < 0.01 compared with control (Con); ^#^ *p* < 0.01 compared with UUO with vehicle (UUO).

TGF-β, one of the most important fibrogenic cytokines, was also upregulated (Figure 4A). The level of phosphorylated ERK was elevated, indicating the activation of ERK signaling in UUO mice (Figure 4B). The upregulation of TGF-β was also inhibited by metformin (Figure 4C). Moreover, the activation of ERK signaling was suppressed by metformin administration (Figure 4D).

### 2.3. ERK Inhibitor PD98059 Inhibited UUO-Induced TGF-β Upregulation and ECM Production

**Figure 5:**
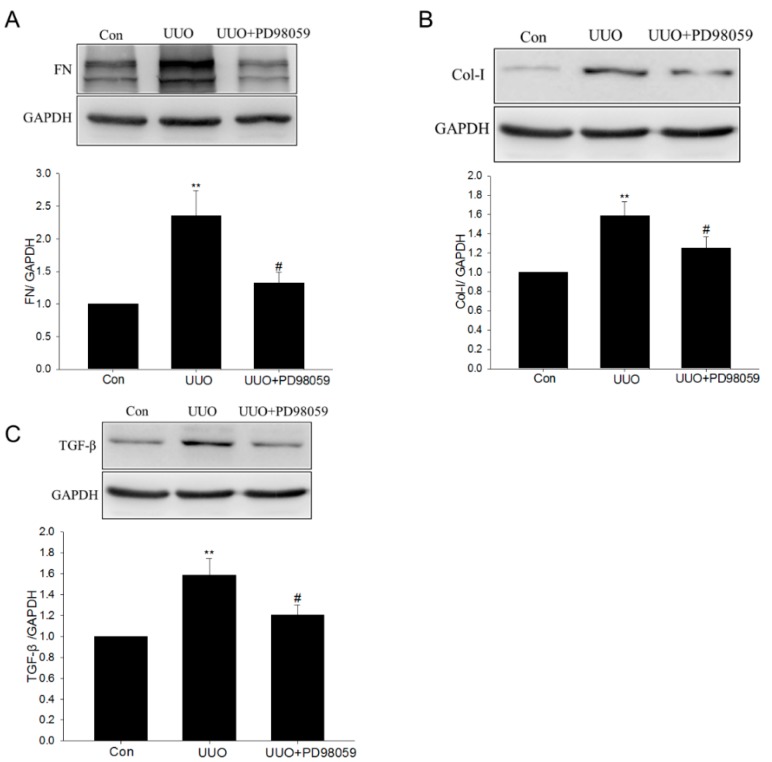
The effect of PD98059 on unilateral ureteral obstruction–induced ECM production and transforming growth factor-β upregulation. (**A**–**C**) The levels of FN, Col-I and TGF-β were analyzed by Western blotting after PD98059 treatment. Data are the mean ± SEM of four animals. ** *p* < 0.01 compared with control (Con); ^#^ *p* < 0.01 compared with UUO with vehicle (UUO).

Western blotting results showed that the upregulation of ECM proteins was suppressed by PD98059 administration in UUO mice (Figure 5A,B). Moreover, the upregulation of TGF-β was also inhibited by PD98059 (Figure 5C).

### 2.4. Renin-Angiotensin System Was Activated in UUO Mice

**Figure 6:**
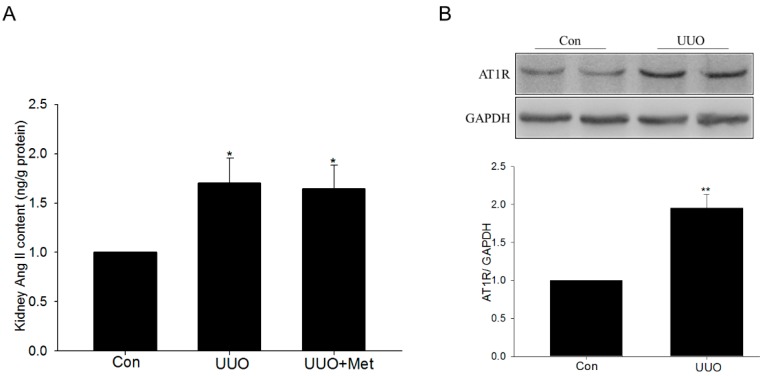
The activation of renin-angiotensin system in UUO mice. (**A**) The level of angiotensin II was detected using an ELISA kit; (**B**) The levels of AT1R were analyzed by Western blotting. Data are the mean ± SEM of six animals. * *p* < 0.05, ** *p* < 0.01 compared with control (Con).

The ELISA result showed that the level of Ang II was increased in obstructed kidney tissues, indicating the activation of RAS in UUO mice. Moreover, metformin administration had no effect on the level of Ang II (Figure 6A). The Western blot results showed that angiotensin II type 1 receptor (AT1R) was also upregulated in obstructed kidney tissues (Figure 6B). Taken together, these results suggested the activation of RAS in UUO mice kidney.

### 2.5. Metformin Inhibited Ang II–Induced Expression of Profibrotic Genes in NRK-49F Cells

**Figure 7:**
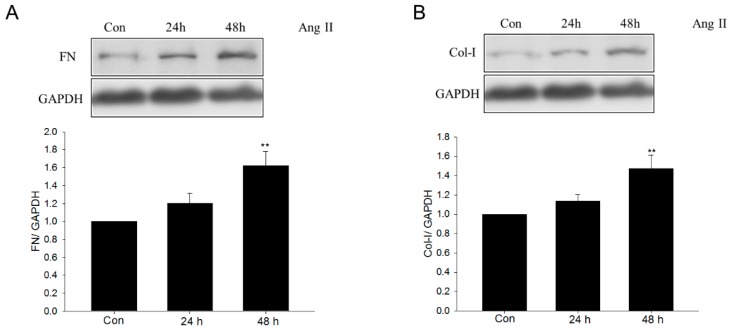

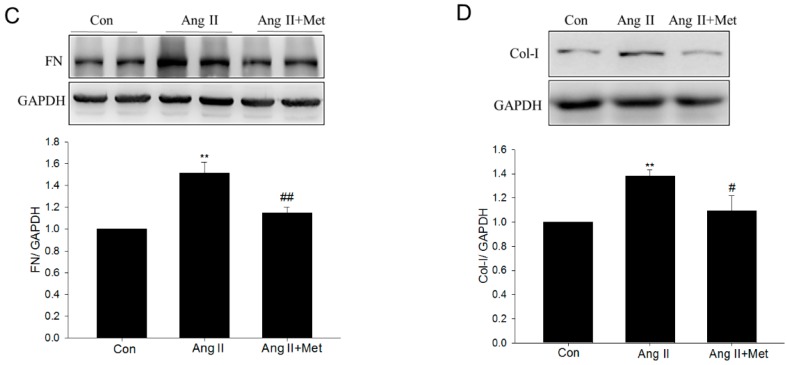
Effect of metformin on angiotensin II–induced ECM production in renal fibroblast NRK-49F cells. (**A**,**B**) The cells were stimulated with 1 μM angiotensin II (Ang II) for 24 or 48 h. Fibronectin (FN) and collagen I (Col-I) were assayed by Western blot analysis; (**C**,**D**) After 48 h of Ang II treatment, with or without 1 h metformin pretreatment. Fibronectin and collagen I were assayed by Western blot analysis. Data are the mean ± SEM of three experiments. ** *p* < 0.01 compared with control (Con); ^#^ *p* < 0.05, ^##^ *p* < 0.01 compared with Ang II only.

To determine whether metformin could reduce profibrotic responses *in vitro*, it was administered to Ang II–treated NRK-49F cells. Western blot analysis showed that Ang II treatment increased fibronectin and collagen I protein levels in NRK-49F cells in a time-dependent manner (Figure 7A,B), confirming the profibrotic effects of Ang II. Pretreatment of metformin for 1 h abolished the Ang II–stimulated upregulation of fibronectin and collagen I (Figure 7C,D).

### 2.6. Metformin Inhibited Ang II–Induced TGF-β Upregulation and ERK Signaling Activation

**Figure 8:**
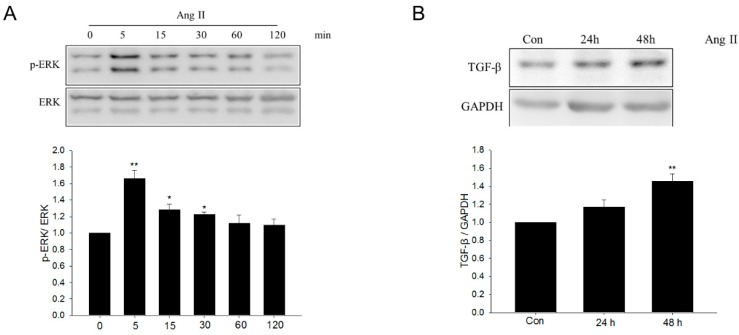

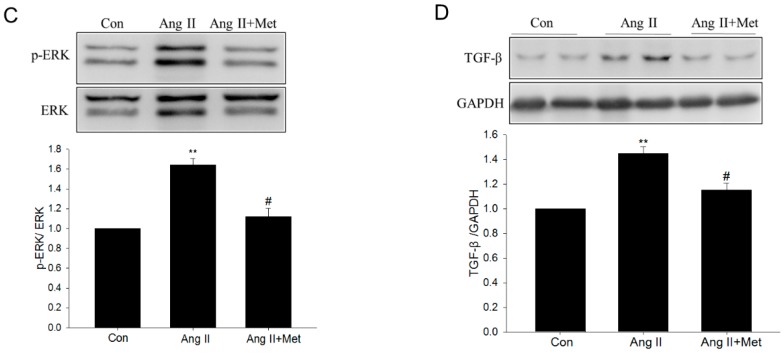
Effect of metformin on angiotensin II–induced transforming growth factor-β upregulation and ERK signaling activation. (**A**,**B**) Cells were treated with angiotensin II (Ang II) for the times indicated. The levels of transforming growth factor-β (TGF-β) and p-ERK were analyzed by Western blotting; (**C**,**D**) TGF-β and p-ERK after metformin treatment, visualized by Western blotting. Data are the mean ± SEM of three experiments. * *p* < 0.05, ** *p* < 0.01 compared with control (Con); ^#^ *p* < 0.05 compared with Ang II only.

**Figure 9:**
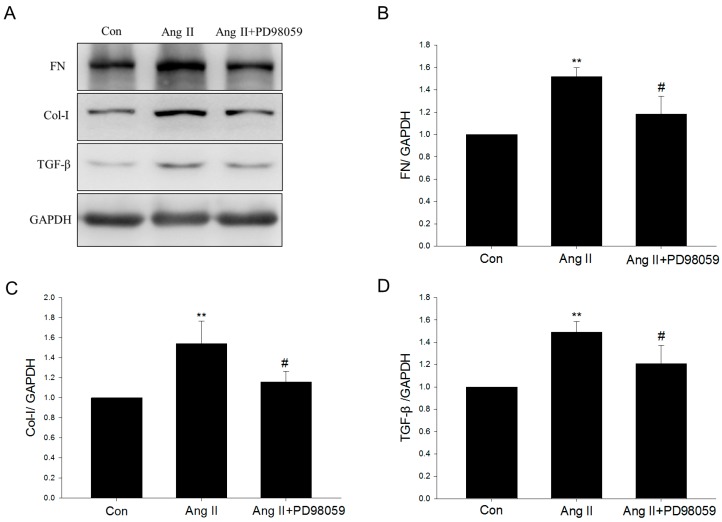
Effect of ERK inhibitor PD98059 on angiotensin II–induced profibrotic effects. (**A**–**D**) After 48 h of Ang II treatment, with or without 1 h ERK inhibitor PD98059 pretreatment. Fibronectin (FN), collagen I (Col-I), and TGF-β were assayed by Western blot analysis. Data are the mean ± SEM of three experiments. * *p* < 0.05, ** *p* < 0.01 compared with control (Con); ^#^ *p* < 0.05 compared with Ang II only.

Western blot analysis showed that Ang II treatment enhanced ERK phosphorylation in NRK-49F cells, indicating the activation of ERK signaling, peaking at 5 min (Figure 8A). Moreover, Ang II treatment increased TGF-β protein levels in NRK-49F cells in a time-dependent manner (Figure 8B). ERK phosphorylation was suppressed by metformin (Figure 8C), suggesting the inhibition of ERK signaling. The upregulation of TGF-β was also suppressed by metformin (Figure 8D). Moreover, ERK signaling inhibitor PD98059 also abolished Ang II–induced profibrotic effects (Figure 9).

## 3. Discussion

Renal fibrosis underlies all forms of end-stage kidney disease, regardless of the primary insult. It is characterized by the excessive accumulation of ECM components after renal injury, which eventually leads to renal failure [4]. Currently, treatment of renal fibrosis is severely limited and often ineffective [3,21]. Kidney transplantation is the only effective option for end-stage renal fibrosis. However, limited organ availability and the high morbidity of transplantation highlight the urgent need for novel strategies to prevent renal fibrosis.

Metformin is the most frequently prescribed antidiabetic drug worldwide. For over half a century, it has been prescribed to patients with type 2 diabetes, yet its underlying mechanism remains largely elusive [12]. Metformin reduces glucose levels and improves insulin sensitivity. Recently, metformin has gained attention for its pleiotropic effects [18]. In this study, we examined the effect of metformin on renal fibrosis.

The animal model used in our study was the UUO model, a well-established model of renal fibrosis. Our results showed that UUO induced significant renal fibrosis, as indicated by the upregulation of ECM proteins and collagen deposition in the kidney. These results suggested that we had successfully built a renal fibrosis model.

Activation of the renin-angiotensin system (RAS) is associated with various deleterious effects, including vasoconstriction, inflammation, hypertrophy, and ECM protein synthesis [22,23,24,25,26]. Ang II, the main effector of the RAS, is a critical mediator of fibrogenesis. The important role of Ang II has been well documented in both diabetic and nondiabetic progressive renal injury. Our ELISA results showed that Ang II was upregulated in the kidney tissue of UUO mice, accompanied by the upregulation of AT1R, suggesting that the RAS had been activated. TGF-β is the key mediator of the profibrotic effects of Ang II [24]. It is a cytokine that plays an essential role in the progression of renal fibrosis in various renal diseases through inducing the synthesis and accumulation of ECM proteins [8]. Ang II induces the activation and transdifferentiation of fibroblasts into scar-forming myofibroblasts, which involves the activation of ERK signaling and the subsequent upregulation of TGF-β [10,23,27,28]. We found that TGF-β was upregulated in UUO mice, and that ERK signaling was also activated, as indicated by the phosphorylation of ERK.

To examine the effect of metformin in renal fibrosis, metformin was administered to UUO mice. As shown by our results, metformin treatment in the UUO mice dramatically attenuated the expression of ECM proteins, including fibronectin and collagen I. The upregulation of TGF-β induced by UUO was abolished by metformin administration. Moreover, ERK phosphorylation was reduced by metformin, indicating that metformin attenuated the activation of ERK signaling. Taken together, these results suggested that metformin attenuated renal fibrosis in UUO mice, accompanied by a decrease in TGF-β and inhibition of the associated ERK signaling pathway.

Resident renal fibroblasts are the most important source of scar-producing myofibroblasts. The activation and proliferation of resident fibroblasts in the kidney result in the enhanced production and excessive deposition of ECM proteins. To confirm the effect of metformin in renal fibrosis, *in vitro* experiments were performed in renal fibroblast NRK-49F cells. The level of ERK phosphorylation was elevated significantly after Ang II treatment, indicating that Ang II induced the activation of ERK signaling. Furthermore, treatment with Ang II induced significant profibrotic effects including the upregulation of TGF-β and excessive production of ECM proteins. Metformin treatment reversed the Ang II–induced ECM overproduction. Moreover, it also abolished the Ang II–induced upregulation of TGF-β, and the activation of ERK signaling. Taken together, these results suggest that metformin could be effective in reversing the profibrotic responses in renal fibroblasts.

## 4. Experimental Section

### 4.1. Materials

Dulbecco’s modified Eagle’s medium (DMEM), recombinant human angiotensin II, metformin, and PD98059 were purchased from Sigma-Aldrich (Saint Louis, MO, USA). Bovine calf serum (BCS) was obtained from Gibco (Grand Island, NY, USA). The BCA Protein Assay Kit was from Shenergy Biocolor BioScience and Technology (Shanghai, China). The anti-fibronectin antibody was obtained from Sigma-Aldrich (Saint Louis, MO, USA), the anti-collagen I antibody and anti-ATIR antibody from Abcam (Cambridge, MA, USA), the anti-TGF-β antibody from Santa Cruz Biotechnologies, Inc. (Santa Cruz, CA, USA), the anti-phospho-ERK and anti-ERK antibodies from Cell Signaling Technology (Danvers, MA, USA), and the anti-GAPDH antibody and an enhanced chemiluminescence (ECL) detection kit and hydrogen peroxide assay kits from Beyotime Institute of Biotechnology (Jiangsu, China). The Ang II ELISA kit was from Westang Biotech (Shanghai, China). Polyvinylidene difluoride (PVDF) membranes were obtained from Millipore (Billerica, MA, USA). The proteinase inhibitor and phosphatase inhibitor were obtained from Roche (Mannheim, Germany). All other chemicals and reagents used were of analytical grade.

### 4.2. UUO Animal Model

Male C57BL/6J mice (20–25 g) were purchased from Shanghai SLAC Laboratory Animal Co., Ltd. (Shanghai, China). Mice were anesthetized with 5% chloral hydrate via intraperitoneal injection. The left ureter was visualized by a flank incision and ligated with 4–0 silk. The sham group underwent the same surgery apart from the left ureter ligation. Over the course of the study, six mice were included in each group and were euthanized three or seven days after surgery. Mice that underwent UUO surgery were randomly divided into three groups: UUO with vehicle (0.9% saline), UUO with metformin (200 mg/kg body weight per day) and UUO with PD98059 (150 mg/kg body weight per day) administered by gavage for seven days. The kidneys were then harvested. All animal experiments were performed according to the Criteria of the Medical Laboratory Animal Administrative Committee of Shanghai and the Guide for Care and Use of Laboratory Animals of Fudan University, and the protocols were approved by the Ethics Committee for Experimental Research, Shanghai Medical College, Fudan University.

### 4.3. Cell Culture

Normal rat kidney fibroblasts (NRK-49F) were purchased from the Institute of Biochemistry and Cell Biology (Shanghai, China). Cells were cultured in DMEM containing 10% BCS, in 5% CO_2_–95% air at 37 °C. They were deprived of serum for 24 h when they reached approximately 80% confluence. The cells were pretreated with 1 mM metformin, 0.1 μM PD98059, or vehicle for 1 h followed by the administration of 1 μM Ang II.

### 4.4. Western Blotting

Protein was isolated from the homogenized frozen kidneys or cell lysate homogenates. Protein concentrations were examined using a BCA Protein Assay Kit according to the manufacturer’s instructions. A standardized amount (30 μg per sample) of total protein was loaded and separated by electrophoresis on a 10% SDS-PAGE gel and then transferred to PVDF membrane. The membrane was blocked with 5% skim milk for 1 h at room temperature with gentle shaking. The membranes were then incubated with the primary antibodies overnight at 4 °C (anti-fibronectin antibody, 1:10,000; anti-collagen I antibody, 1:500; anti-AT1R antibody, 1:500; anti-phospho-ERK antibody, 1:1000; anti-ERK antibody, 1:1000; anti-TGF-β antibody, 1:500; and anti-GAPDH antibody, 1:3000). The membranes were then washed three times with Tris-buffered saline (TBS) with 0.1% Tween and incubated with horseradish peroxidase–conjugated secondary antibodies for 1 h at room temperature. After another three washes with TBS/Tween, the hybridizing bands were developed using the ECL detection kit according to the manufacturer’s instructions. Protein bands were visualized by the GE chemiluminescence system for the required time. Bands were normalized using GAPDH or ERK.

### 4.5. Measurement of the Kidney Concentration of Ang II

The concentration of Ang II was measured using commercially available enzyme immunoassay kits (Westang Biotech, Shanghai, China). The procedures were performed according to the manufacturer’s instructions. The Ang II concentration was calculated from the standard curve constructed with standard solutions.

### 4.6. Histological Examination

The kidneys were fixed in 10% neutral buffered formalin, and then embedded in paraffin. Sections were collected and prepared. Slides of paraffinized tissue sections were deparaffinized, rehydrated, and washed in distilled water. Sections were stained with Masson’s trichrome. Histological changes were observed at 200× optical magnification.

### 4.7. Statistical Analyses

Data are presented as means ± SEM with the statistical analysis performed using one-way analysis of variance with *post hoc* analysis using Tukey’s multiple comparison test. The differences between two groups were compared using Student’s *t* test. *p* < 0.05 was considered to indicate a statistically significant difference between mean values.

## 5. Conclusions

In summary, our study showed that metformin attenuated renal fibrosis effectively both *in vitro* and *in vivo*, and that these effects were associated with the downregulation of TGF-β and the inhibition of the ERK signaling pathway. Given its high safety and cheapness, we propose that metformin could potentially be used clinically to treat renal fibrosis.
